# [μ-*N*,*N*,*N*′,*N*′-Tetra­kis(2-pyridyl­meth­yl)butane-1,4-diamine]­bis­[dinitratocadmium(II)]

**DOI:** 10.1107/S1600536810034549

**Published:** 2010-09-04

**Authors:** Mark Bartholomä, Hoi Cheung, Jon Zubieta

**Affiliations:** a Department of Chemistry, Syracuse University, Syracuse, New York 13244, USA

## Abstract

The title dinuclear cadmium complex, [Cd_2_(NO_3_)_4_(C_28_H_32_N_6_)], is located on an inversion center. The unique Cd^II^ ion displays a 5 + 2 coordination. A distorted square-pyramidal geometry is formed by the dipicolyl­amine group of the ligand *via* the N atoms in a meridional fashion and two O atoms of the nitrate ligands with short Cd—O distances. The coordination is completed by two loosely bound O atoms of the nitrate ligands.

## Related literature

For crystallographic data of tetra­kis­(pyridin-2-yl-meth­yl)alkyl-diamine compounds, see: Fujihara *et al.* (2004 ▶); Mambanda *et al.* (2007 ▶). For the superoxide dismutase activity of iron complexes, see: Tamura *et al.* (2000 ▶). For dinuclear Pt complexes of similar ligands, see: Ertürk *et al.* (2007 ▶). For the use of the dipicolyl­amine moiety for binding of the *M*(CO)_3_ core (*M* = Re, ^99*m*^Tc), see: Bartholomä *et al.* (2009 ▶). For crystal structures closely related to the title compound, see: Bartholomä *et al.* (2010*a* ▶,*b* ▶,*c* ▶,*d* ▶)
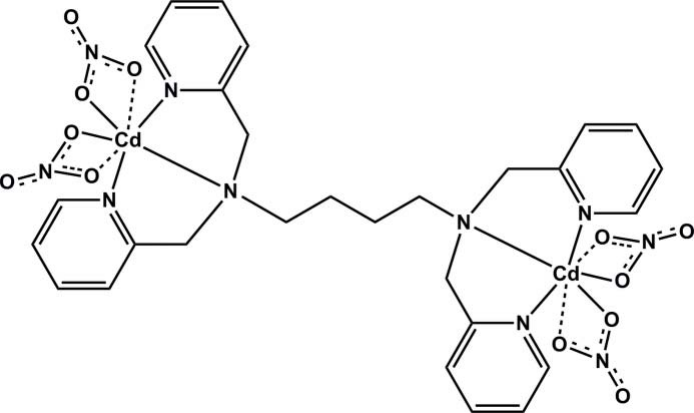


## Experimental

### 

#### Crystal data

[Cd_2_(NO_3_)_4_(C_28_H_32_N_6_)]
*M* *_r_* = 925.44
Triclinic, 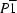

*a* = 8.0548 (8) Å
*b* = 8.7010 (8) Å
*c* = 13.2566 (13) Å
α = 107.488 (2)°
β = 96.767 (2)°
γ = 104.631 (2)°
*V* = 838.30 (14) Å^3^
*Z* = 1
Mo *K*α radiation
μ = 1.35 mm^−1^
*T* = 90 K
0.48 × 0.30 × 0.08 mm

#### Data collection

Bruker SMART APEX diffractometer
Absorption correction: multi-scan (*SADABS*; Bruker, 1998 ▶) *T*_min_ = 0.564, *T*_max_ = 0.900
8382 measured reflections
4043 independent reflections
3912 reflections with *I* > 2σ(*I*)
*R*_int_ = 0.020

#### Refinement

*R*[*F* ^2^ > 2σ(*F* ^2^)] = 0.030
*wR*(*F* ^2^) = 0.069
*S* = 1.15
4043 reflections
235 parameters
H-atom parameters constrained
Δρ_max_ = 1.01 e Å^−3^
Δρ_min_ = −0.50 e Å^−3^

### 

Data collection: *SMART* (Bruker, 1998 ▶); cell refinement: *SAINT* (Bruker, 1998 ▶); data reduction: *SAINT*; program(s) used to solve structure: *SHELXS97* (Sheldrick, 2008 ▶); program(s) used to refine structure: *SHELXL97* (Sheldrick, 2008 ▶); molecular graphics: *DIAMOND* (Brandenburg & Putz, 1999 ▶); software used to prepare material for publication: *SHELXTL* (Sheldrick, 2008 ▶).

## Supplementary Material

Crystal structure: contains datablocks I, global. DOI: 10.1107/S1600536810034549/lh5105sup1.cif

Structure factors: contains datablocks I. DOI: 10.1107/S1600536810034549/lh5105Isup2.hkl

Additional supplementary materials:  crystallographic information; 3D view; checkCIF report

## Figures and Tables

**Table 1:** Selected bond lengths (Å)

Cd1—N2	2.250 (2)
Cd1—N3	2.251 (2)
Cd1—O5	2.279 (2)
Cd1—O1	2.322 (2)
Cd1—O2	2.588 (2)
Cd1—O4	2.687 (2)
Cd1—N1	2.427 (2)

**Fig. 1.**
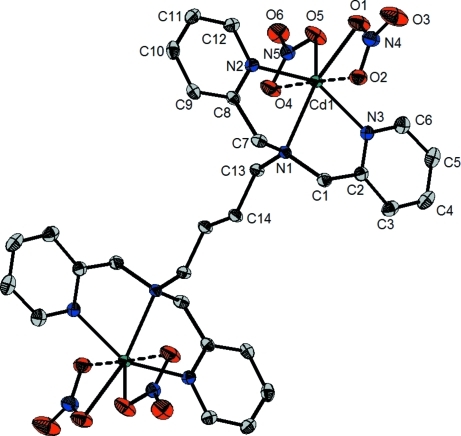
The crystal structure of the title complex. The displacement ellipsoids are drawn at 50% probability level. Hydrogen atoms are omitted for clarity. Unlabeled atoms are related by the symmetry code (-x, -y + 2, -z + 1).

## supplementary crystallographic information

### Comment

The described ligand *N^1^*,*N^1^*,*N^4^*,*N^4^*-tetrakis(pyridin-2-ylmethyl)butane-1,4-diamine has been used as starting material in the hydrothermal synthesis of metal-organic transition metal/molybdateoxide frameworks in the principal author's laboratory (Bartholomä, unpublished results). The title complex was prepared as part of a series with different cadmium and copper salts to study the coordination properties of the ligand with these metals without the interaction of metaloxide clusters (Bartholomä, 2010*b*,c,*d*).

Another crystalline species of a monomeric dinuclear cadmium complex using the corresponding acetate salt as metal source has been reported by our group (Bartholomä, 2010*a*). In the cadmium acetate structure, the Cd—N distances of the pyridine N atoms are slightly longer [2.313 (3) Å and 2.379 (3) Å] whereas the distance of the tertiary nitrogen atom is with marginally shorter [2.405 (3)] Å when compared to the distances of the title complex.

The dipicolylamine moiety has originally been developed in our laboratory as metal chelating entity for binding of the *M*(CO)_3_ core (*M* = Re,^99^*^m^*Tc) for radiopharmaceutical purposes. However, a different coordination mode has been observed for the *M*(CO)_3_ core in which the dipicolylamine metal chelate is coordinated in a facial manner (Bartholomä, 2009).

Crystal structures of the ligands *N^1^*,*N^1^*,*N^3^*,*N^3^*-tetrakis(2-pyridiniomethyl)-1,3-diaminopropane and *N^1^*,*N^1^*,*N^4^*,*N^4^*-tetrakis(pyridin-2-ylmethyl)butane-1,4-diamine have been described recently (Fujihara, 2004; Mambanda, 2007). Superoxide dismutase activity of iron(II) complexes of *N^1^*,*N^1^*,*N^3^*,*N^3^*-tetrakis(2-pyridiniomethyl)-1,3-diaminopropane and related ligands has been investigated by Tamura *et al.* (2000). Studies on the thermodynamic and kinetic behaviour of the reaction of platinum(II) complexes of higher ligand homologues with chloride have been performed by Ertürk *et al.* (2007).

### Experimental

*N^1^*,*N^1^*,*N^4^*,*N^4^*-tetrakis(pyridin-2-ylmethyl)butane-1,4-diamine. An amount of 1.00 g (11.34 mmol) 1,4-diaminobutane was dissolved in 30 ml anhydrous dichloroethane under an inert atmosphere (argon) followed by the addition of 4.55 ml (47.65 mmol) pyridine-2-carboxaldehyde. The mixture was stirred for 15 min at r.t. and then cooled with an ice bath prior to the portionwise addition of 14.43 g (68.06 mmol) sodium triacetoxyborohydride (gas evolution, exothermic reaction). The reaction was stirred overnight allowing the temperature slowly to rise to room temperature. The reaction was quenched by the dropwise addition of saturated sodium bicarbonate solution and stirring was continued until the gas evolution ceased. The mixture was separated and the organic layer was further washed with saturated sodium bicarbonate solution, water and brine. The organic phase was dried with anhydrous sodium sulfate, filtered and the solvent removed under reduced pressure. The crude reaction mixture was then purified by silica gel column chromatography starting with chloroform and increasing gradient to chloroform:methanol 10:1 (*v*/*v*). Yield: 4.02 g (78%). ^1^H NMR (CDCl_3_): *δ* = 8.40 (m, 4H), 7.51 (m, 4H), 7.39 (d, J = 7.81 Hz, 4H), 7.02 (m, 4H), 3.67 (s, 8H), 2.39 (m, 4H), 1.42 (m, 4H) p.p.m..

Synthesis of metal complex. To 2 ml of an aqueous solution of cadmium nitrate, two equivalents (50 mg, 0.11 mmol) of *N^1^*,*N^1^*,*N^4^*,*N^4^*-tetrakis(pyridin-2-ylmethyl)butane-1,4-diamine in 2 ml methanol were added followed by the addition of 2 ml *N*,*N*-dimethylformamide. Single crystals were obtained after a week by slow evaporation of the solvents at room temperature.

### Refinement

All the H atoms were placed in idealized positions and refined using the riding model approximation with *C*—H_aryl_ = 0.95 Å, *C*—H_methyl_ = 0.98Å and *C*—H_methylene_ = 0.99Å and with *U*_iso_(H) = 1.5*U*_eq_(C_methyl_) and 1.2*U*_eq_(C_methylene/aryl_).

### Crystal data

**Table:**

[Cd_2_(NO_3_)_4_(C_28_H_32_N_6_)]	*Z* = 1
*M**_r_* = 925.44	*F*(000) = 462
Triclinic, *P*1	*D*_x_ = 1.833 Mg m^−^^3^
Hall symbol: -P 1	Mo *K*α radiation, λ = 0.71073 Å
*a* = 8.0548 (8) Å	Cell parameters from 5752 reflections
*b* = 8.7010 (8) Å	θ = 2.5–28.4°
*c* = 13.2566 (13) Å	µ = 1.35 mm^−^^1^
α = 107.488 (2)°	*T* = 90 K
β = 96.767 (2)°	Plate, colourless
γ = 104.631 (2)°	0.48 × 0.30 × 0.08 mm
*V* = 838.30 (14) Å^3^	

### Data collection

**Table:**

Bruker SMART APEX diffractometer	4043 independent reflections
Radiation source: fine-focus sealed tube	3912 reflections with *I* > 2σ(*I*)
graphite	*R*_int_ = 0.020
Detector resolution: 512 pixels mm^-1^	θ_max_ = 28.1°, θ_min_ = 1.7°
φ and ω scans	*h* = −10→10
Absorption correction: multi-scan (*SADABS*; Bruker, 1998)	*k* = −11→11
*T*_min_ = 0.564, *T*_max_ = 0.900	*l* = −16→17
8382 measured reflections	

### Refinement

**Table:**

Refinement on *F*^2^	Primary atom site location: structure-invariant direct methods
Least-squares matrix: full	Secondary atom site location: difference Fourier map
*R*[*F*^2^ > 2σ(*F*^2^)] = 0.030	Hydrogen site location: inferred from neighbouring sites
*wR*(*F*^2^) = 0.069	H-atom parameters constrained
*S* = 1.15	*w* = 1/[σ^2^(*F*_o_^2^) + (0.0286*P*)^2^ + 0.8284*P*] where *P* = (*F*_o_^2^ + 2*F*_c_^2^)/3
4043 reflections	(Δ/σ)_max_ = 0.001
235 parameters	Δρ_max_ = 1.01 e Å^−^^3^
0 restraints	Δρ_min_ = −0.50 e Å^−^^3^

### Special details

**Table:**

Geometry. All e.s.d.'s (except the e.s.d. in the dihedral angle between two l.s. planes) are estimated using the full covariance matrix. The cell e.s.d.'s are taken into account individually in the estimation of e.s.d.'s in distances, angles and torsion angles; correlations between e.s.d.'s in cell parameters are only used when they are defined by crystal symmetry. An approximate (isotropic) treatment of cell e.s.d.'s is used for estimating e.s.d.'s involving l.s. planes.
Refinement. Refinement of *F*^2^ against ALL reflections. The weighted *R*-factor *wR* and goodness of fit *S* are based on *F*^2^, conventional *R*-factors *R* are based on *F*, with *F* set to zero for negative *F*^2^. The threshold expression of *F*^2^ > σ(*F*^2^) is used only for calculating *R*-factors(gt) *etc*. and is not relevant to the choice of reflections for refinement. *R*-factors based on *F*^2^ are statistically about twice as large as those based on *F*, and *R*- factors based on ALL data will be even larger.

### Fractional atomic coordinates and isotropic or equivalent isotropic displacement parameters (Å^2^)

**Table:**

	*x*	*y*	*z*	*U*_iso_*/*U*_eq_	
Cd1	0.37059 (2)	0.85996 (2)	0.212593 (14)	0.01675 (6)	
O1	0.4785 (3)	0.7640 (3)	0.05839 (16)	0.0335 (5)	
O2	0.2104 (3)	0.6092 (3)	0.03615 (16)	0.0270 (4)	
O3	0.3515 (3)	0.5592 (3)	−0.09358 (19)	0.0511 (7)	
O4	0.5248 (3)	1.1197 (3)	0.39807 (18)	0.0347 (5)	
O5	0.6551 (3)	1.0276 (3)	0.27055 (17)	0.0347 (5)	
O6	0.7985 (3)	1.2505 (3)	0.41010 (18)	0.0356 (5)	
N1	0.0990 (3)	0.8167 (2)	0.27756 (16)	0.0158 (4)	
N2	0.2297 (3)	1.0238 (3)	0.15892 (16)	0.0186 (4)	
N3	0.3643 (3)	0.6590 (3)	0.28768 (16)	0.0189 (4)	
N4	0.3458 (3)	0.6419 (3)	−0.00139 (19)	0.0269 (5)	
N5	0.6610 (3)	1.1361 (3)	0.36162 (18)	0.0221 (4)	
C1	0.0530 (3)	0.6391 (3)	0.2732 (2)	0.0207 (5)	
H1A	−0.0030	0.5639	0.1979	0.025*	
H1B	−0.0335	0.6213	0.3190	0.025*	
C2	0.2108 (3)	0.5907 (3)	0.31117 (19)	0.0194 (5)	
C3	0.1947 (4)	0.4684 (3)	0.3606 (2)	0.0245 (5)	
H3A	0.0869	0.4226	0.3787	0.029*	
C4	0.3379 (4)	0.4149 (3)	0.3827 (2)	0.0276 (6)	
H4A	0.3289	0.3310	0.4158	0.033*	
C5	0.4951 (4)	0.4839 (3)	0.3566 (2)	0.0273 (6)	
H5	0.5944	0.4475	0.3706	0.033*	
C6	0.5034 (4)	0.6068 (3)	0.3099 (2)	0.0238 (5)	
H6A	0.6109	0.6562	0.2928	0.029*	
C7	−0.0241 (3)	0.8446 (3)	0.19778 (19)	0.0192 (5)	
H7A	−0.1265	0.8629	0.2287	0.023*	
H7B	−0.0672	0.7419	0.1322	0.023*	
C8	0.0606 (3)	0.9957 (3)	0.16622 (18)	0.0175 (5)	
C9	−0.0369 (3)	1.0924 (3)	0.13757 (19)	0.0203 (5)	
H9	−0.1557	1.0729	0.1446	0.024*	
C10	0.0413 (4)	1.2182 (3)	0.0984 (2)	0.0229 (5)	
H10	−0.0244	1.2841	0.0772	0.028*	
C11	0.2153 (4)	1.2464 (3)	0.0906 (2)	0.0225 (5)	
H11	0.2712	1.3318	0.0641	0.027*	
C12	0.3059 (3)	1.1477 (3)	0.1222 (2)	0.0208 (5)	
H12	0.4260	1.1675	0.1180	0.025*	
C13	0.1306 (3)	0.9396 (3)	0.38864 (18)	0.0167 (4)	
H13A	0.1702	1.0550	0.3854	0.020*	
H13B	0.2283	0.9253	0.4341	0.020*	
C14	−0.0244 (3)	0.9281 (3)	0.44558 (19)	0.0194 (5)	
H14A	−0.1265	0.9347	0.3994	0.023*	
H14B	−0.0579	0.8180	0.4571	0.023*	

### Atomic displacement parameters (Å^2^)

**Table:**

	*U*^11^	*U*^22^	*U*^33^	*U*^12^	*U*^13^	*U*^23^
Cd1	0.01544 (9)	0.01864 (10)	0.01966 (10)	0.00647 (6)	0.00744 (6)	0.00900 (7)
O1	0.0278 (10)	0.0318 (11)	0.0282 (10)	−0.0031 (8)	0.0117 (8)	0.0001 (8)
O2	0.0228 (9)	0.0307 (10)	0.0304 (10)	0.0077 (8)	0.0088 (8)	0.0137 (8)
O3	0.0489 (14)	0.0507 (15)	0.0297 (12)	−0.0048 (12)	0.0180 (10)	−0.0073 (10)
O4	0.0253 (10)	0.0371 (11)	0.0373 (11)	0.0056 (9)	0.0163 (9)	0.0063 (9)
O5	0.0217 (10)	0.0412 (12)	0.0302 (11)	0.0043 (9)	0.0097 (8)	−0.0004 (9)
O6	0.0222 (10)	0.0322 (11)	0.0377 (12)	−0.0033 (8)	0.0008 (8)	0.0031 (9)
N1	0.0168 (9)	0.0142 (9)	0.0159 (9)	0.0048 (7)	0.0058 (7)	0.0036 (7)
N2	0.0203 (10)	0.0182 (10)	0.0184 (10)	0.0058 (8)	0.0056 (8)	0.0073 (8)
N3	0.0200 (10)	0.0176 (10)	0.0196 (10)	0.0061 (8)	0.0052 (8)	0.0063 (8)
N4	0.0271 (12)	0.0264 (11)	0.0255 (11)	0.0053 (9)	0.0100 (9)	0.0072 (9)
N5	0.0207 (10)	0.0242 (11)	0.0249 (11)	0.0093 (9)	0.0069 (8)	0.0105 (9)
C1	0.0185 (11)	0.0171 (11)	0.0258 (12)	0.0041 (9)	0.0082 (9)	0.0065 (9)
C2	0.0253 (12)	0.0146 (11)	0.0150 (11)	0.0036 (9)	0.0053 (9)	0.0018 (9)
C3	0.0313 (14)	0.0178 (12)	0.0194 (12)	0.0017 (10)	0.0049 (10)	0.0046 (9)
C4	0.0402 (16)	0.0172 (12)	0.0217 (12)	0.0043 (11)	−0.0005 (11)	0.0077 (10)
C5	0.0318 (14)	0.0210 (12)	0.0268 (13)	0.0094 (11)	−0.0017 (11)	0.0068 (10)
C6	0.0228 (12)	0.0215 (12)	0.0246 (12)	0.0059 (10)	0.0021 (10)	0.0062 (10)
C7	0.0161 (11)	0.0226 (12)	0.0180 (11)	0.0046 (9)	0.0046 (9)	0.0067 (9)
C8	0.0190 (11)	0.0190 (11)	0.0137 (10)	0.0065 (9)	0.0043 (8)	0.0035 (9)
C9	0.0194 (11)	0.0241 (12)	0.0181 (11)	0.0107 (9)	0.0047 (9)	0.0047 (9)
C10	0.0312 (14)	0.0218 (12)	0.0167 (11)	0.0142 (10)	0.0018 (10)	0.0039 (9)
C11	0.0302 (13)	0.0173 (11)	0.0192 (12)	0.0049 (10)	0.0045 (10)	0.0071 (9)
C12	0.0215 (12)	0.0201 (12)	0.0201 (12)	0.0040 (9)	0.0048 (9)	0.0078 (9)
C13	0.0159 (11)	0.0167 (10)	0.0152 (10)	0.0023 (8)	0.0048 (8)	0.0036 (8)
C14	0.0184 (11)	0.0199 (11)	0.0196 (12)	0.0059 (9)	0.0081 (9)	0.0047 (10)

### Geometric parameters (Å, °)

**Table:**

Cd1—N2	2.250 (2)	C3—C4	1.381 (4)
Cd1—N3	2.251 (2)	C3—H3A	0.9500
Cd1—O5	2.279 (2)	C4—C5	1.389 (4)
Cd1—O1	2.322 (2)	C4—H4A	0.9500
Cd1—O2	2.588 (2)	C5—C6	1.379 (4)
Cd1—O4	2.687 (2)	C5—H5	0.9500
Cd1—N1	2.427 (2)	C6—H6A	0.9500
O1—N4	1.273 (3)	C7—C8	1.521 (3)
O2—N4	1.254 (3)	C7—H7A	0.9900
O3—N4	1.231 (3)	C7—H7B	0.9900
O4—N5	1.243 (3)	C8—C9	1.387 (3)
O5—N5	1.276 (3)	C9—C10	1.391 (4)
O6—N5	1.233 (3)	C9—H9	0.9500
N1—C1	1.477 (3)	C10—C11	1.382 (4)
N1—C7	1.479 (3)	C10—H10	0.9500
N1—C13	1.485 (3)	C11—C12	1.381 (4)
N2—C8	1.342 (3)	C11—H11	0.9500
N2—C12	1.351 (3)	C12—H12	0.9500
N3—C6	1.344 (3)	C13—C14	1.530 (3)
N3—C2	1.345 (3)	C13—H13A	0.9900
C1—C2	1.514 (4)	C13—H13B	0.9900
C1—H1A	0.9900	C14—C14^i^	1.528 (5)
C1—H1B	0.9900	C14—H14A	0.9900
C2—C3	1.396 (4)	C14—H14B	0.9900
			
N2—Cd1—N3	148.10 (8)	C3—C4—C5	119.9 (2)
N2—Cd1—O5	103.49 (8)	C3—C4—H4A	120.1
N3—Cd1—O5	103.16 (8)	C5—C4—H4A	120.1
N2—Cd1—O1	98.56 (8)	C6—C5—C4	118.3 (3)
N3—Cd1—O1	103.09 (8)	C6—C5—H5	120.9
O5—Cd1—O1	79.90 (7)	C4—C5—H5	120.9
N2—Cd1—N1	74.12 (7)	N3—C6—C5	122.2 (3)
N3—Cd1—N1	74.27 (7)	N3—C6—H6A	118.9
O5—Cd1—N1	139.43 (7)	C5—C6—H6A	118.9
O1—Cd1—N1	140.62 (7)	N1—C7—C8	112.38 (19)
N4—O1—Cd1	100.89 (15)	N1—C7—H7A	109.1
N5—O5—Cd1	105.38 (15)	C8—C7—H7A	109.1
C1—N1—C7	112.91 (19)	N1—C7—H7B	109.1
C1—N1—C13	113.20 (19)	C8—C7—H7B	109.1
C7—N1—C13	113.10 (19)	H7A—C7—H7B	107.9
C1—N1—Cd1	104.04 (14)	N2—C8—C9	121.2 (2)
C7—N1—Cd1	103.73 (14)	N2—C8—C7	117.4 (2)
C13—N1—Cd1	108.92 (13)	C9—C8—C7	121.2 (2)
C8—N2—C12	119.4 (2)	C8—C9—C10	119.3 (2)
C8—N2—Cd1	116.61 (16)	C8—C9—H9	120.4
C12—N2—Cd1	123.99 (17)	C10—C9—H9	120.4
C6—N3—C2	119.9 (2)	C11—C10—C9	119.4 (2)
C6—N3—Cd1	123.75 (17)	C11—C10—H10	120.3
C2—N3—Cd1	116.38 (17)	C9—C10—H10	120.3
O3—N4—O2	121.4 (2)	C12—C11—C10	118.4 (2)
O3—N4—O1	120.3 (2)	C12—C11—H11	120.8
O2—N4—O1	118.3 (2)	C10—C11—H11	120.8
O6—N5—O4	122.7 (2)	N2—C12—C11	122.3 (2)
O6—N5—O5	120.0 (2)	N2—C12—H12	118.8
O4—N5—O5	117.3 (2)	C11—C12—H12	118.8
N1—C1—C2	112.7 (2)	N1—C13—C14	116.85 (19)
N1—C1—H1A	109.0	N1—C13—H13A	108.1
C2—C1—H1A	109.0	C14—C13—H13A	108.1
N1—C1—H1B	109.0	N1—C13—H13B	108.1
C2—C1—H1B	109.0	C14—C13—H13B	108.1
H1A—C1—H1B	107.8	H13A—C13—H13B	107.3
N3—C2—C3	120.8 (2)	C14^i^—C14—C13	110.4 (2)
N3—C2—C1	117.8 (2)	C14^i^—C14—H14A	109.6
C3—C2—C1	121.2 (2)	C13—C14—H14A	109.6
C4—C3—C2	119.0 (3)	C14^i^—C14—H14B	109.6
C4—C3—H3A	120.5	C13—C14—H14B	109.6
C2—C3—H3A	120.5	H14A—C14—H14B	108.1
			
N2—Cd1—O1—N4	−82.99 (18)	Cd1—O5—N5—O6	−176.1 (2)
N3—Cd1—O1—N4	73.42 (18)	Cd1—O5—N5—O4	4.4 (3)
O5—Cd1—O1—N4	174.73 (19)	C7—N1—C1—C2	152.9 (2)
N1—Cd1—O1—N4	−7.6 (2)	C13—N1—C1—C2	−77.0 (3)
N2—Cd1—O5—N5	74.64 (18)	Cd1—N1—C1—C2	41.1 (2)
N3—Cd1—O5—N5	−87.67 (18)	C6—N3—C2—C3	1.3 (3)
O1—Cd1—O5—N5	171.11 (18)	Cd1—N3—C2—C3	−178.25 (17)
N1—Cd1—O5—N5	−6.6 (2)	C6—N3—C2—C1	−173.2 (2)
N2—Cd1—N1—C1	147.56 (15)	Cd1—N3—C2—C1	7.2 (3)
N3—Cd1—N1—C1	−28.13 (14)	N1—C1—C2—N3	−35.6 (3)
O5—Cd1—N1—C1	−120.12 (16)	N1—C1—C2—C3	149.8 (2)
O1—Cd1—N1—C1	63.46 (19)	N3—C2—C3—C4	−1.6 (4)
N2—Cd1—N1—C7	29.25 (14)	C1—C2—C3—C4	172.8 (2)
N3—Cd1—N1—C7	−146.43 (15)	C2—C3—C4—C5	0.6 (4)
O5—Cd1—N1—C7	121.57 (16)	C3—C4—C5—C6	0.7 (4)
O1—Cd1—N1—C7	−54.85 (19)	C2—N3—C6—C5	0.0 (4)
N2—Cd1—N1—C13	−91.45 (15)	Cd1—N3—C6—C5	179.57 (19)
N3—Cd1—N1—C13	92.86 (15)	C4—C5—C6—N3	−1.0 (4)
O5—Cd1—N1—C13	0.9 (2)	C1—N1—C7—C8	−154.2 (2)
O1—Cd1—N1—C13	−175.55 (14)	C13—N1—C7—C8	75.6 (2)
N3—Cd1—N2—C8	−5.2 (3)	Cd1—N1—C7—C8	−42.3 (2)
O5—Cd1—N2—C8	−151.19 (16)	C12—N2—C8—C9	−0.5 (3)
O1—Cd1—N2—C8	127.22 (17)	Cd1—N2—C8—C9	178.50 (17)
N1—Cd1—N2—C8	−13.12 (16)	C12—N2—C8—C7	174.4 (2)
N3—Cd1—N2—C12	173.73 (17)	Cd1—N2—C8—C7	−6.6 (3)
O5—Cd1—N2—C12	27.8 (2)	N1—C7—C8—N2	36.1 (3)
O1—Cd1—N2—C12	−53.8 (2)	N1—C7—C8—C9	−149.0 (2)
N1—Cd1—N2—C12	165.9 (2)	N2—C8—C9—C10	1.4 (4)
N2—Cd1—N3—C6	−175.28 (17)	C7—C8—C9—C10	−173.3 (2)
O5—Cd1—N3—C6	−29.3 (2)	C8—C9—C10—C11	−1.2 (4)
O1—Cd1—N3—C6	53.2 (2)	C9—C10—C11—C12	0.1 (4)
N1—Cd1—N3—C6	−167.4 (2)	C8—N2—C12—C11	−0.7 (4)
N2—Cd1—N3—C2	4.3 (3)	Cd1—N2—C12—C11	−179.60 (18)
O5—Cd1—N3—C2	150.30 (16)	C10—C11—C12—N2	0.9 (4)
O1—Cd1—N3—C2	−127.20 (17)	C1—N1—C13—C14	−62.6 (3)
N1—Cd1—N3—C2	12.17 (16)	C7—N1—C13—C14	67.4 (3)
Cd1—O1—N4—O3	178.6 (2)	Cd1—N1—C13—C14	−177.82 (17)
Cd1—O1—N4—O2	−1.2 (3)	N1—C13—C14—C14^i^	−175.1 (2)

Symmetry codes: (i) −*x*, −*y*+2, −*z*+1.

## References

[bb1] Bartholomä, M., Cheung, H., Darling, K. & Zubieta, J. (2010*d*). *Acta Cryst.* E**66**, m1201–m1202. 10.1107/S1600536810034513 PMC2983384 21587361

[bb2] Bartholomä, M., Cheung, H. & Zubieta, J. (2010*a*). *Acta Cryst.* E**66**, m1195–m1196. 10.1107/S1600536810034550 PMC2983136 21587357

[bb3] Bartholomä, M., Cheung, H. & Zubieta, J. (2010b). *Acta Cryst.* E**66**, m1198. 10.1107/S1600536810034537 PMC2983387 21587359

[bb4] Bartholomä, M., Cheung, H. & Zubieta, J. (2010*c*). *Acta Cryst.* E**66**, m1199–m1200. 10.1107/S1600536810034501 PMC2983221 21587360

[bb5] Bartholomä, M., Valliant, J., Maresca, K. P., Babich, J. & Zubieta, J. (2009). *Chem. Commun.* **5**, 473–604. 10.1039/b814903h 19283279

[bb6] Brandenburg, K. & Putz, H. (1999). *DIAMOND* Crystal Impact GbR, Bonn, Germany.

[bb7] Bruker (1998). *SMART*, *SAINT* and *SADABS* Bruker AXS Inc., Madison, Wisconsin, USA.

[bb8] Ertürk, H., Hofmann, A., Puchta, R. & van Eldik, R. (2007). *Dalton Trans.* pp. 2295–2301. 10.1039/b700770c 17534490

[bb9] Fujihara, T., Saito, M. & Nagasawa, A. (2004). *Acta Cryst.* E**60**, o1126–o1128.

[bb10] Mambanda, A., Jaganyi, D. & Munro, O. Q. (2007). *Acta Cryst.* C**63**, o676–o680. 10.1107/S0108270107049293 17989495

[bb11] Sheldrick, G. M. (2008). *Acta Cryst.* A**64**, 112–122. 10.1107/S0108767307043930 18156677

[bb12] Tamura, M., Urano, Y., Kikuchi, K., Higuchi, T., Hirobe, M. & Nagano, T. (2000). *J. Organomet. Chem.* **611**, 586–592. 10.1248/cpb.48.1514 11045460

